# Immediate vs. Delayed Implant Placement Following Alveolar Ridge Procedures with Xeno-Hybrid Bovine Bone Graft: A Retrospective Cohort with Operator-Level Comparison

**DOI:** 10.3390/diagnostics16081231

**Published:** 2026-04-20

**Authors:** Marius Meier, Pascal Grün, Tim Schiepek, Pina Jankowski, Anna Bandura, Sebastian Fitzek, Flora Turhani, Dritan Turhani

**Affiliations:** 1 Center for Oral and Maxillofacial Surgery, Department of Dentistry, Faculty of Medicine and Dentistry, Danube Private University, Steiner Landstraße 124, 3500 Krems, Austria; 2 Clinical Application of Artificial Intelligence in Dentistry (CAAID) Group, Department of Dentistry, Faculty of Medicine and Dentistry, Danube Private University, Steiner Landstraße 124, 3500 Krems an der Donau, Austria; 3 Medical Image Analysis & Artificial Intelligence (MIAAI) Group, Faculty of Medicine and Dentistry, Danube Private University, Steiner Landstrasse 124, 3500 Krems, Austria

**Keywords:** immediate implant placement, alveolar ridge preservation, alveolar ridge reconstruction, xeno-hybrid bovine graft, early implant loss, rehabilitation timelines, operator level, retrospective cohort

## Abstract

**Background:** In everyday practice, we observed an increased number of complications with a xeno-hybrid bovine graft in challenging cases, prompting a systematic review of timing strategies and the impact of the operator. **Objective:** To compare early safety and rehabilitation timelines for immediate versus delayed implant placement after alveolar ridge procedures with a xeno-hybrid bovine graft, and to examine operator-level effects. **Materials and Methods:** Single center retrospective cohort (Danube Private University, Krems, Austria; January 2021–October 2023). Consecutive patients undergoing alveolar ridge preservation (ARP) or reconstruction (ARR; conservative protocol without meshes or rigid frameworks) with a xeno-hybrid bovine graft and subsequent implant were included. Strata: ARP, ARR, ARP with immediate implant placement (ARP+II), ARR with immediate implant placement (ARR+II). Primary endpoint: early implant loss < 12 months after the index surgery. Secondary endpoints: days to implant exposure and days to definitive prosthesis. **Results:** We analyzed 158 coded interventions (ARP 33; ARR 16; ARP+II 32; ARR+II 77). Early implant loss was uncommon (7/158; 4.4%) and occurred only with immediate implant placement (ARP+II 6.3%; ARR+II 6.5%); no early failures occurred in delayed strata. Immediate implant placement accelerated rehabilitation (exposure: 358/364 vs. 144/162 days; prosthesis: 406/419 vs. 196/204 days; both *p* < 0.0001). After adjustment for treatment base, timing, and age, no independent operator level effect on early loss was detected. **Conclusions:** In this university cohort using a xeno-hybrid bovine graft, early implant loss was rare and confined to immediate implant placement, which nonetheless shortened the pathway to exposure and restoration by ~5–7 months. Differences across providers were explained by case selection and protocol choice after adjustment. **Clinical Significance:** With appropriate case selection and surgical execution, immediate implant placement after ARP/ARR can reduce treatment time by ~5–7 months without a clear increase in early failure within the limits of this cohort; treatment protocol and case triage are the main levers of early outcome.

## 1. Introduction

Tooth extraction leads to dimensional remodeling of the alveolar ridge. After unassisted healing, about 29–63% of ridge width (2.5–4.6 mm) and 11–22% of ridge height (0.8–1.5 mm) are typically lost within roughly six months, most of it during the first 3–6 months [1,2]. These reductions compromise prosthetically driven implant positioning and may necessitate secondary augmentation at implant placement.

Alveolar ridge preservation (ARP) and alveolar ridge reconstruction (ARR) aim to attenuate this physiologic collapse. Systematic reviews and meta-analyses show that, compared with spontaneous healing over 3–6 months, ARP prevents ≈2 mm of horizontal loss and ≈1–2 mm of vertical loss at mid-buccal/lingual landmarks, particularly when socket grafting is combined with resorbable coverage [3,4,5]. Beyond dimensional benefits, early ridge management reduces the frequency and magnitude of ancillary grafting and can streamline rehabilitation [1,6].

From a safety perspective, ARP is associated with low short-term event rates. Typical soft-tissue issues are limited to wound dehiscence or membrane exposure; pooled estimates in ARP/GBR settings place exposure in the low-to-mid teens, particularly with extensive flaps or non-resorbable barriers [7]. Early implant loss after ARP is uncommon and broadly comparable to non-grafted controls, with meta-analyses and cohort studies reporting high short-term survival [5,8,9]. In contrast, ARR carries a higher soft-tissue complication burden, with overall healing complications around 16–20% [10,11,12]. Even so, when complications are prevented or managed early, implant survival in regenerated sites remains high and broadly comparable to that in native bone [13].

By contrast, deferring augmentation until implant placement often confronts a more atrophic ridge, increasing reliance on horizontal/vertical guided bone regeneration (GBR) with membranes/meshes or sinus augmentation procedures with greater technical complexity and a meaningful risk of complications (exposure, dehiscence, infection) that can jeopardize vertical gain and prolong care pathways [14,15,16,17]. Recent vertically oriented augmentation trials and network meta-analyses likewise report that techniques achieving greater vertical gain tend to show higher healing-complication incidence, with membrane/mesh exposure as a pivotal determinant of outcome [18,19].

Against this biologic and procedural backdrop, implant timing remains debated. Aggregated comparisons generally show similar survival for immediate vs. delayed placement. In contrast, immediate strategies shorten the path to functional restoration, provided that case selection and soft-tissue management are rigorous [11,16]. Longer-term analyses suggest that context (e.g., risk profile, aesthetic zone) may influence survival beyond 5–6 years, underscoring the importance of real-world studies stratified by treatment type and operating environment [20].

Material choice is integral to this calculus. Bovine-derived xenografts, including xeno-hybrid composites (bovine mineral matrices reinforced with polymer/collagen), are widely used in ARP/ARR; recent clinical and materials reports describe favorable osteoconductive integration and site-specific remodeling for xeno-hybrid grafts, while acknowledging that high-level randomized evidence for xeno-hybrids is still developing [18,19].

Beyond general considerations of graft selection, this project was motivated by an internal signal at our center: in routine practice, a xeno-hybrid bovine graft appeared to be associated with more frequent complications in clinically demanding situations, particularly in cases with multi-socket or segmental defects requiring ridge reconstruction, reduced residual bone volume affecting primary stability, compromised soft-tissue conditions affecting tension-free closure, and simultaneous augmentation with immediate implant placement; this concern was first raised around lateral-window sinus procedures [21]. While reports on xeno-hybrid materials in maxillofacial reconstruction are emerging, comparative data for alveolar ridge procedures (ARP/ARR) remain sparse. To our knowledge, no prior cohort has evaluated immediate versus delayed implant placement after ARP/ARR using a xeno-hybrid bovine graft while also examining operator-level effects. This gap, together with our in-house signal, underpins the present study.

Within a university care setting, where operator level (OMFS specialists vs. supervised students and general dentists) co-varies with case complexity, the practical question is whether immediate vs. delayed implant placement after ARP/ARR with a xeno-hybrid bovine graft differs in early complications and time-to-rehabilitation, and whether any observed differences persist after accounting for treatment selection and case-mix. We therefore conducted a single-center retrospective cohort to: (1) compare early severe complications (implant loss in the first year) across immediate vs. delayed strategies following ARP/ARR with a xeno-hybrid bovine graft; (2) explore operator-level effects in adjusted analyses, hypothesizing accelerated rehabilitation without a material increase in early events and attenuation of operator-level differences after adjustment; and (3) quantify days to implant exposure and days to prosthetic restoration.

## 2. Material and Methods

### 2.1. Study Design and Setting

We conducted a single-center retrospective cohort study at the Danube Private University (DPU) in Krems, Austria, within a university-based dental and maxillofacial surgery (OMFS) service. The cohort reflects everyday clinical practice and was intentionally broad to capture a real-world case-mix. We included all consecutive patients treated during the study period who underwent ridge preservation or reconstruction with a specific xeno-hybrid bovine bone graft and subsequently received a dental implant at the exact center. A curated Master dataset and operator-level source sheets formed the analytical basis; for operator-focused analyses, the Master sheet and operator sheets were not co-analyzed together to avoid duplication.

Study period. Consecutive eligibility from January 2021 through October 2023.

Reporting standard. The study follows STROBE guidance for observational cohort studies [22].

Ethics. The study protocol was reviewed and approved by the Danube Private University Ethics Committee, Krems an der Donau, Austria (DPU-EK/066, approved 30 April 2024). Given the retrospective design and the use of de-identified records, the requirement for individual informed consent was waived. The study was conducted in accordance with the Declaration of Helsinki and applicable data-protection regulations (GDPR), and reporting follows STROBE guidance for observational cohort studies.

### 2.2. Data Sources and Cohort Assembly

Eligibility. All consecutive patients managed at DPU Krems between January 2021 and October 2023 who received a specific xeno-hybrid bovine graft for ridge procedures (ARP or ARR) and subsequently received an implant at the exact center; the cohort was deliberately heterogeneous to mirror real-world care.

Assembly and flow. Source sheets were harmonized and merged into a Master dataset. For operator-level analyses, operator sheets were analyzed separately from the Master to prevent duplicate counting.

Uncoded/NA. Records lacking definitive treatment coding were retained only for cohort flow/baseline counts and excluded from all inferential comparisons (odds ratios, timeline tests) and from operator-stratified analyses.

### 2.3. Definitions and Group Coding

Index surgery. The surgical appointment initiates the recorded pathway; all time-to-event measures start here.

Treatment strata (mutually exclusive).

- ARP: single extraction socket grafted immediately after extraction to mitigate dimensional change using resorbable barriers only; implant delayed.
- ARR: augmentation of the alveolar ridge involving multiple sockets or segments, performed either as a staged procedure on a healed ridge or immediately after or during tooth extraction when multiple adjacent sockets are grafted; performed without titanium meshes or rigid frameworks and using resorbable barriers only; implant placement delayed unless coded as ARR+II.
- ARP+II: ARP with immediate implant placement at index surgery.
- ARR+II: ARR with immediate implant placement at index surgery.

Immediate vs. delayed. Immediate = implant at index surgery; Delayed = implant after healing. This definition applies to both ARP and ARR; when ARR is performed immediately after or during extraction, implant insertion in the same session is coded as ARR+II, whereas implant insertion after healing is coded as ARR.

Operator level. Operators were categorized based on formal training background and clinical responsibility within the university setting. The OMFS group comprised board-certified specialists in oral and maxillofacial surgery. The non-OMFS group included general dentists and dental students performing procedures under supervision within a structured clinical teaching environment. This grouping reflects differences in formal surgical training and level of autonomy rather than individual case volume. Case allocation was not randomized and followed routine clinical workflow, with supervision and case complexity influencing assignment. Rows without an operator label were excluded from operator-level analyses. Handling of Uncoded/NA. Kept only for cohort flow/baseline; excluded from all hypothesis tests and operator analyses.

### 2.4. Outcomes

Primary outcome. The primary endpoint was early implant loss occurring <12 months after the index surgery. Other early postoperative issues (e.g., dehiscence, exposure, infection) were recorded when available but were not part of the primary endpoint and are reported descriptively only. Although soft-tissue complications such as dehiscence or membrane exposure were not part of the primary endpoint, their occurrence may represent precursor events contributing to early implant loss, particularly in immediate placement scenarios.

Secondary outcomes (time to rehabilitation, days).

1. Days to implant exposure: calendar days from index surgery to uncovering/second-stage.
2. Days to prosthetic restoration: calendar days from index surgery to delivery of the definitive prosthesis.

For time-to-event analyses, listwise exclusion was applied within the endpoint if required dates were missing; no imputation beyond recorded dates.

### 2.5. Covariates

Pre-specified covariates for adjusted analyses were: age at operation (years, continuous); immediate implant (II) (binary; implant placed at the index surgery); treatment base (ARP vs. ARR) to distinguish socket-level from staged multi-segment contexts; and operator level (OMFS vs. non-OMFS) for operator-focused models.

Comorbidities and medications. We also recorded patient comorbidities (e.g., diabetes, cardiovascular disease) and regular medications (e.g., antiplatelets, anticoagulants, antiresorptives). These variables were summarized descriptively but were not included in adjusted models due to heterogeneous documentation, sparse categories, and a low number of primary outcome events, which could increase the risk of overfitting and destabilize estimates in rare-event settings.

### 2.6. Data Curation and Missing Data

Harmonization and parsing. Source sheets were standardized; clinical free text on adverse events was parsed into the binary complication endpoint using pre-specified rules.

Uncoded/NA. Retained for flow/baseline only; excluded from (i) OR contrasts, (ii) timeline hypothesis tests, and (iii) operator-stratified analyses.

Operator labels. Rows without an operator label were excluded only from operator-level analyses.

Zero-event cells. Anticipated in some strata and handled using continuity-corrected estimators and penalized regression.

Endpoint-specific missingness. Time-to-event analyses used within-endpoint listwise exclusion when required dates were missing; no imputation beyond recorded values.

### 2.7. Statistical Analysis

Due to the retrospective design, no a priori sample size calculation was performed. Primary outcome. Group rates with Wilson 95% confidence intervals; odds ratios vs ARP estimated with Haldane–Anscombe continuity correction for zero cells. Uncoded/NA excluded.

Timelines. Kruskal–Wallis tests across coded treatment strata; Benjamini–Hochberg–adjusted pairwise Mann–Whitney tests where specified. Uncoded/NA excluded.

Operator models. Ridge-penalized logistic regression adjusted for treatment base, immediate implant placement, and age; operator coded OMFS vs. non-OMFS.

### 2.8. Software and Reporting Standards

All analyses were performed in Python 3.10.9 using pandas, NumPy, SciPy, statsmodels, matplotlib, plotly, and python-docx according to the pre-specified statistical plan in the analyst’s report. Reporting follows the STROBE guideline for observational cohort studies [22].

## 3. Results

### 3.1. Overall Cohort

We analyzed 183 interventions performed at a single university center between January 2021 and October 2023. Of these, 158 interventions were fully coded for inferential analyses, and 25 were retained for baseline/flow only (Uncoded/NA). Among coded cases, the treatment mix comprised ARP (n = 33), ARR (n = 16), ARP+II (n = 32), and ARR+II (n = 77), corresponding to 49 delayed placements (ARP, ARR) and 109 immediate implant placements (ARP+II, ARR+II). Age was available for 158 patients (mean 57.4 ± 12.7 years), with slightly higher means in ARR and ARR+II, consistent with a heterogeneous case mix.

**Figure 1:**
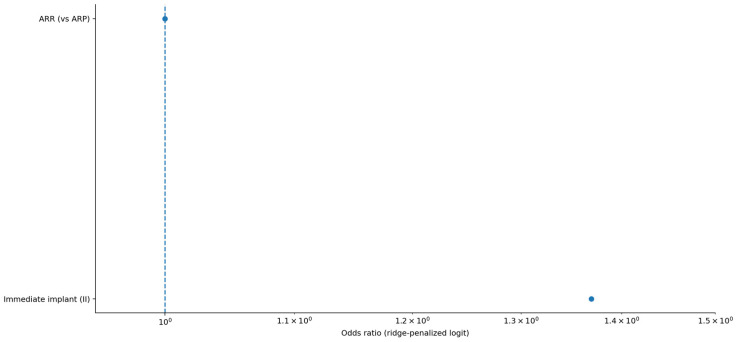
Ridge-penalized odds ratios for early implant loss. Dots indicate odds ratios; the dashed vertical line indicates OR = 1.

**Figure 2:**
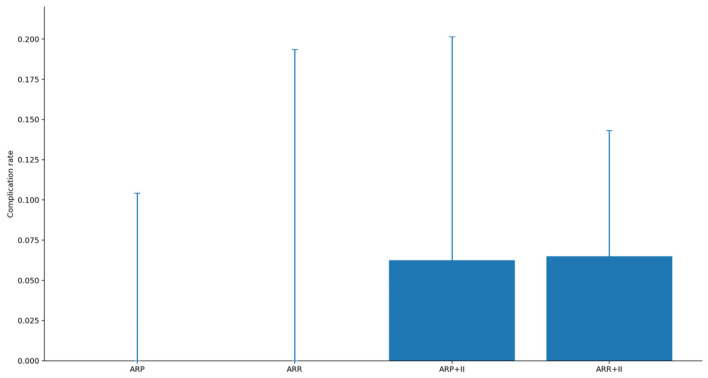
Early implant loss rates by treatment (Wilson 95% confidence intervals).

**Figure 3:**
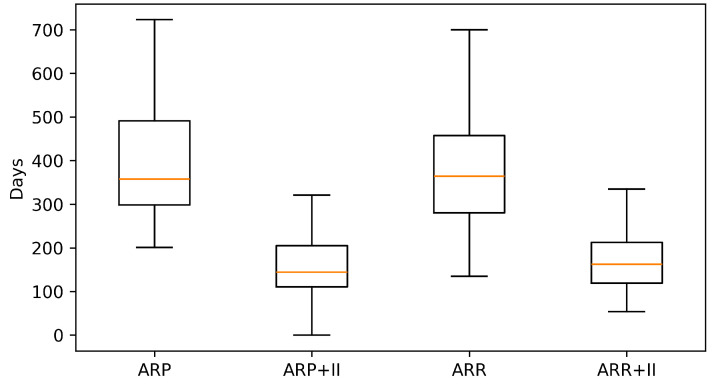
Days from surgery to implant exposure by treatment.

**Figure 4:**
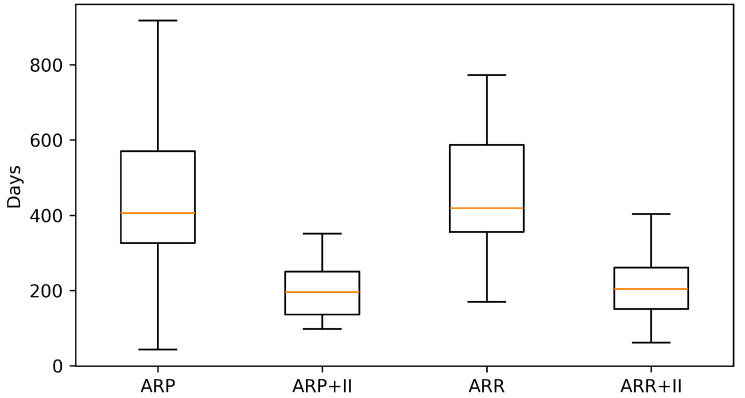
Days from surgery to definitive prosthetic restoration by treatment.

Across the coded cohort, early implant loss < 12 months was uncommon (7/158; 4.4%) and occurred only in immediate implant placement strata; no early failures were recorded in delayed groups. The distribution of treatments and baseline characteristics is summarized in Table 1. The adjusted effects on early implant loss are illustrated in Figure 1 and summarized in Table 2 Complication rates across treatment strata are shown in Figure 2 and Table 3a, with continuity-corrected odds ratios presented in Table 3b. Immediate implant placement shortened rehabilitation: time to implant exposure and to definitive prosthesis were roughly five to seven months faster than delayed strategies (full distributions summarized below). The distribution of time to implant exposure and prosthetic restoration across treatment strata is shown in Figure 3 and Figure 4, and Table 4.

#### Operator-Level Comparison

Rows with missing operator labels were excluded from operator-only tests.

### 3.2. Stratified by Timing (Immediate vs. Delayed)

When grouped by timing, immediate implant placement (ARP+II, ARR+II combined) accounted for 109/158 cases and included all seven early losses. In contrast, delayed placement (ARP, ARR combined; 49/158 cases) showed no early implant loss in this cohort. Timelines favored immediate strategies: median time to exposure was 144 days [IQR 111–205] for ARP+II and 162 [119–212] for ARR+II versus 358 [298–491] for ARP and 364 [280–458] for ARR (Kruskal–Wallis *p* < 0.0001). The pattern was mirrored for definitive prosthesis: 196 [136–250] and 204 [151–261] days for ARP+II and ARR+II versus 406 [326–570] and 419 [356–587] days for ARP and ARR (*p* < 0.0001).

### 3.3. Stratified by Treatment Base (ARP vs. ARR)

Within ARP-based care, delayed ARP showed no early loss and substantially longer timelines than ARP+II, which achieved the expected acceleration without a detectable early-failure penalty in our small-event setting. Within ARR-based care (performed conservatively without meshes or rigid frameworks and including multi-socket contexts at or around extraction), delayed ARR likewise showed no early loss, whereas ARR+II carried the majority of events (5/77; 6.5 percent) alongside the same time advantage seen for immediate implant placement. These patterns are consistent with the combined demands of simultaneous augmentation and implant insertion.

### 3.4. Operator Context

**Figure 5:**
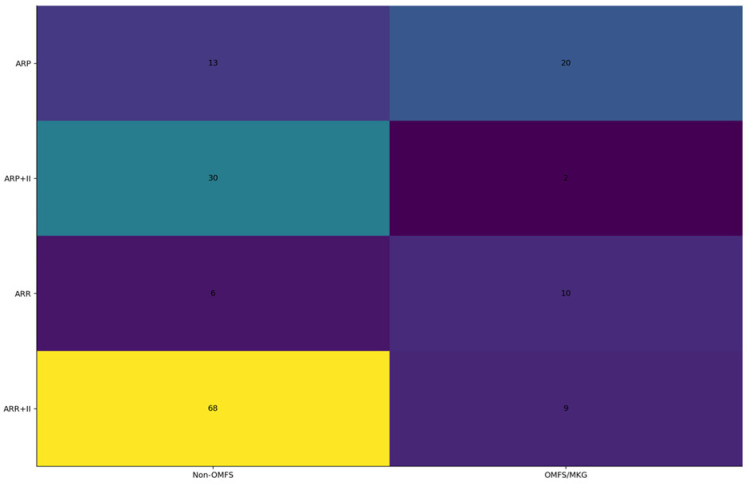
Treatment-by-operator heatmap. Cell shading reflects counts; darker cells indicate larger counts.

**Figure 6:**
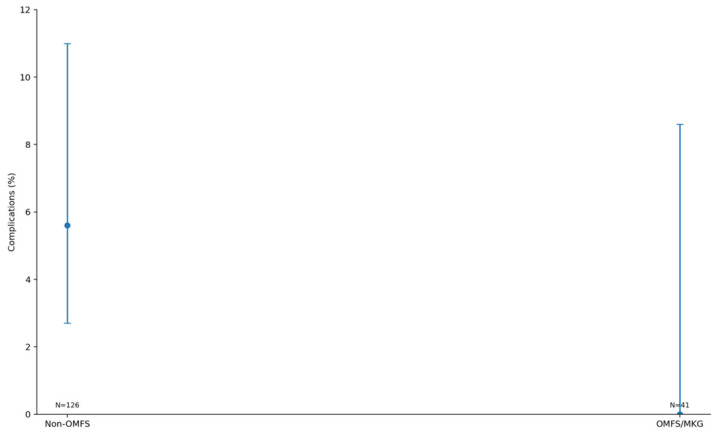
Early implant loss rate by operator (Wilson 95% confidence intervals). Points indicate rates; vertical bars indicate 95% confidence intervals.

**Figure 7:**
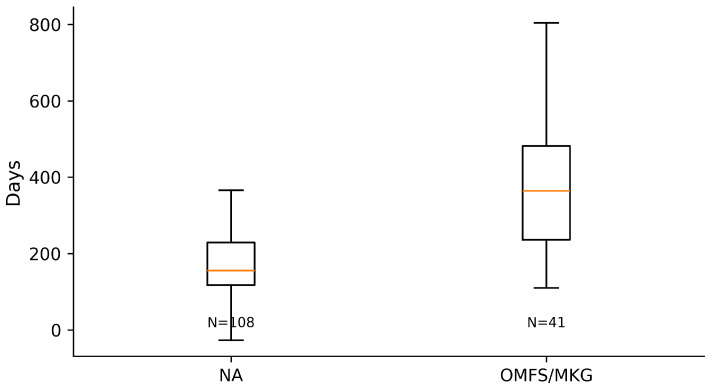
Days from surgery to implant exposure by operator.

**Figure 8:**
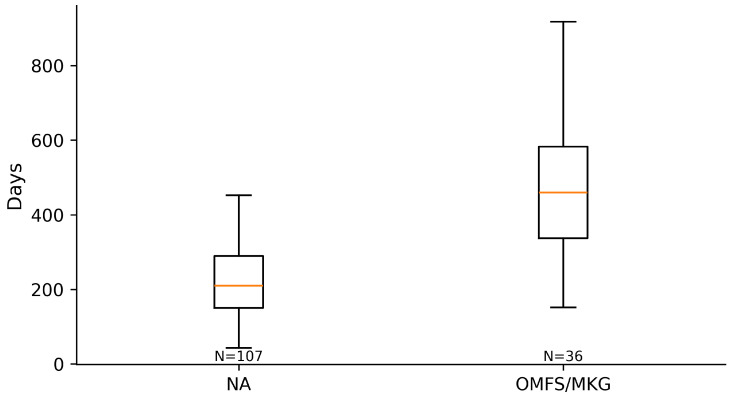
Days from surgery to definitive prosthetic restoration by operator.

**Figure 9:**
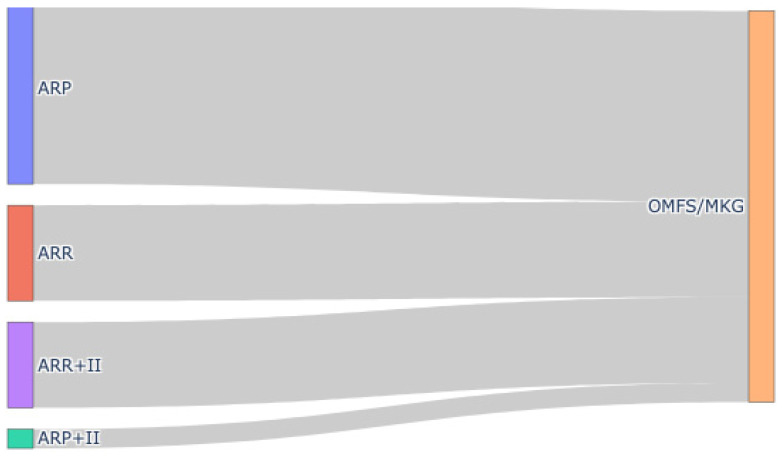
Treatment-to-operator flow diagram. Colors identify treatment strata; band width is proportional to case count.

Treatment allocation differed markedly by operator level, with OMFS specialists more often staging ARP and non-OMFS providers more often performing ARR+II. No early failures occurred in OMFS cases, and all seven arose in the non-OMFS stream; however, in ridge-penalized logistic regression adjusted for treatment base, immediate implant placement, and age, we found no independent operator-level effect on early implant loss (immediate placement adjusted OR ≈ 1.37; confidence intervals suppressed due to quasi-separation). The adjusted model results are illustrated in Figure 1 and detailed in Table 3. Taken together, these findings support confounding by indication and protocol as the primary explanation for the unadjusted distribution of events. The allocation of treatments by operator level is shown in Figure 5 and summarized in Table 5, with the association statistics reported in Table 6. Operator-specific complication rates are illustrated in Figure 6 and summarized in Table 7, with pairwise comparisons presented in Table 8. The adjusted operator model is reported in Table 9. Timeline differences by operator level are visualized in Figure 7 and Figure 8, with summary statistics provided in Table 10 and pairwise tests in Table 11 and Table 12. The relationship between treatment allocation and operator level is further illustrated in Figure 9.

## 4. Discussion

We observed seven early implant losses (7/158; 4.4%), with events confined to immediate implant placement strata and none in delayed groups or among OMFS cases, warranting a structured assessment of timing, operator context, and case selection.

In our cohort, early implant loss was rare overall and confined to immediate placement strata (2/32 ARP+II; 5/77 ARR+II); no early failures occurred after delayed placement (ARP/ARR) or among OMFS cases. The absence of early failures in delayed groups may also reflect differences in case complexity rather than timing alone. Immediate implant placement shortened time to implant exposure from roughly ~12 months to ~5–6 months, and time to definitive restoration from ~13–14 months to ~6–7 months. The absence of an independent operator-level effect after adjustment is likely multifactorial. First, all procedures were performed within a structured university environment with standardized surgical protocols for ARP/ARR and implant placement, which may reduce inter-operator variability. Second, non-OMFS providers operated under supervision, which likely mitigated differences in technical execution and clinical decision-making. Third, treatment allocation was not random: OMFS specialists more frequently performed staged approaches (ARP), whereas non-OMFS providers more often performed immediate ARR+II cases, reflecting differences in case selection and workflow. This pattern introduces confounding by indication, whereby more complex or time-sensitive cases are clustered within specific operator groups. After adjustment for treatment base, immediate placement, and age, the attenuation of the operator effect suggests that protocol choice and case-mix, rather than intrinsic operator performance, are the primary drivers of early outcomes in this setting. However, given the small number of events, these findings should be interpreted cautiously, and the absence of a detected operator-level effect should not be interpreted as evidence of absence given the limited statistical power.

Because ARR at our clinic is deliberately conservative (no meshes or rigid frameworks), potential pathways to early loss most likely reflect the combined demands of simultaneous augmentation and implant insertion, primary stability, tension free soft tissue closure, and graft stability, rather than mesh related exposure. The clustering of loss in immediate strata fits this rationale, while the lack of an adjusted signal underscores the role of case selection.

These findings should be interpreted cautiously. With seven early losses, precision is limited; absence of significance does not imply equivalence. Comorbidities and medications were recorded but not modeled due to heterogeneity and sparsity in documentation, leaving room for residual confounding. In practice, a low threshold for staging (ARP with delayed implant) remains sensible when surgical demands or soft-tissue conditions are marginal; when immediate implant placement is chosen, emphasis on primary stability, flap design, and early wound protection is key.

The xeno-hybrid graft is biologically plausible and operatively practical, with emerging clinical data indicating reliable osteoconductive behavior [16,20]. The polymer/collagen reinforcement of xeno-hybrid grafts may influence handling characteristics, graft cohesion, and early soft-tissue interaction, potentially affecting stability in simultaneous augmentation settings. These properties may be particularly relevant in demanding cases requiring immediate implant placement, where primary stability and soft-tissue sealing are critical. Our data do not allow conclusions regarding material-specific performance after adjustment. However, the rare-event context limits precision, and the study was not powered to detect small differences. Importantly, this study did not include a parallel comparator material; inferences about the xeno-hybrid graft are therefore context-specific to our timing and protocol choices rather than comparative claims of superiority or inferiority. Nevertheless, given the in-house signal that motivated this analysis, attention to flap design, wound protection, and primary stability is warranted whenever simultaneous augmentation and implantation are combined.

The descriptive absence of early failures in OMFS cases, together with longer timelines in that stream, is most consistent with staging choices and case triage, a view supported by the adjusted models, which did not identify an operator-level risk signal. In supervised university settings, protocol selection outweighs title. When indications are matched and procedures are structured, non-OMFS providers can achieve early outcomes comparable to specialists while still leveraging the time advantages of immediate implant placement in suitable cases.

The retrospective design and the relatively small number of early failures (n = 7) limit precision. Confidence intervals are wide, so non-significant results should not be taken as evidence of equivalence. A small subset lacked definitive treatment coding and was therefore excluded from inference. Although we recorded comorbidities and regular medications, heterogeneous documentation and sparse categories prevented their use in adjusted models, leaving room for residual confounding. Follow-up focused on early outcomes; longer-term biologic and esthetic endpoints and patient-reported measures were not captured systematically.

This study has several limitations. First, the retrospective design limits causal inference and is susceptible to selection bias. Second, the number of early implant loss events was low (n = 7), restricting statistical power and precision of effect estimates. Third, no a priori sample size calculation was performed due to the retrospective nature of the study. Fourth, the absence of a comparator graft material precludes any conclusions regarding material-specific performance. Fifth, treatment allocation was not randomized and likely reflects confounding by indication and case selection. Finally, comorbidities and medications were not included in adjusted models due to heterogeneous documentation, leaving room for residual confounding.

Although Uncoded/NA cases were excluded from inferential analyses, no systematic clustering suggesting differential risk was observed; however, exclusion of these cases may still introduce bias.

In appropriately selected cases, immediate implant placement after ARP/ARR can enable exposure and restoration ~5–6 months and ~6–7 months sooner, respectively, without a clear increase in early failure within the limits of this cohort. Where soft-tissue conditions are marginal or surgical demands are high, a low threshold for staging (ARP with delayed implant) remains prudent. Across providers, protocol choice and triage seem to have the most impact.

Prospective, multicenter cohorts with standardized capture of systemic risk factors, esthetic outcomes, and patient-reported measures are needed. Matched or randomized studies comparing xeno-hybrid vs conventional xenografts in both staged and simultaneous workflows would clarify whether any material-specific differences emerge under controlled soft-tissue conditions and longer follow-up.

## 5. Conclusions

In this real-world university cohort using a xeno-hybrid bovine graft, early implant loss was uncommon and occurred only in immediate implant placement strata. No early failures were observed after delayed placement or among OMFS cases. After adjustment for treatment base, timing, and age, we found no independent effect of operator level. Immediate implant placement consistently shortened the path to implant exposure and to definitive restoration by roughly five to seven months, a clinically meaningful difference.

Taken together, these data suggest that protocol selection and case triage, rather than material choice alone, are the primary drivers of early outcomes. When soft tissue conditions are limited or surgical demands are high, a staged approach with ARP and delayed implant placement remains a prudent default. When immediate implant placement is chosen, careful attention to primary stability, flap design, and early wound protection is essential. Although our analyses did not isolate a material-specific penalty for the xeno-hybrid bovine graft, the rare event context limits precision, and confirmation in larger prospective cohorts is warranted. Future studies should standardize the capture of systemic risk and patient-centered outcomes, and directly compare xeno-hybrid and conventional xenografts in both staged and simultaneous workflows.

## Figures and Tables

**Table 1:** Sample characteristics by treatment (Master dataset).

Treatment	N	Age_N	Age_Mean	Age_SD
**ARP**	33	33	53.243	11.675
**ARR**	16	16	63.937	13.388
**ARP+II**	32	32	55.947	13.586
**ARR+II**	77	77	58.462	12.112
**Uncoded/NA**	25	0		
**Total**	183	158	57.417	12.702

**Table 2:** Ridge-penalized logistic regression (OR; CIs omitted).

Term	Coefficient	OR	Note
**const**	−3.157	0.043	Ridge-penalized logit; confidence intervals omitted due to quasi-separation.
**treatment_base_ARR**	**0.0**	**1.0**	**Ridge-penalized logit; confidence intervals omitted due to quasi-separation.**
**immediate_ii**	0.314	1.369	Ridge-penalized logit; confidence intervals omitted due to quasi-separation.

**Table 3:** Early implant loss by treatment: (a) rates with Wilson 95% confidence intervals and (b) continuity-corrected odds ratios versus ARP.

(**a**) Rates by treatment (Wilson 95% confidence intervals)							
**Treatment**	**N**		**Complications**	**Rate**	**CI95_Lo**		**CI95_Hi**
**ARP**	33		0	0.0	0.0		0.104
**ARR**	16		0	0.0	0.0		0.194
**ARP+II**	32		2	0.062	0.017		0.201
**ARR+II**	77		5	0.065	0.028		0.143
**Uncoded/NA**	25		0	0.0	0.0		0.133
(**b**) Continuity-corrected odds ratios versus ARP							
**Comparison**		**OR**		**CI95_Lo**		**CI95_Hi**	
**ARR vs. ARP**		2.03		0.039		106.931	
**ARP+II vs. ARP**		5.492		0.253		118.984	
**ARR+II vs. ARP**		5.083		0.273		94.606	

**Table 4:** Rehabilitation timelines by treatment (median [IQR]; Kruskal-Wallis).

Metric	Treatment	N	Median [IQR]
**days_to_freileg**	ARP	32	358 [298, 491]
**days_to_freileg**	ARP+II	30	144 [111, 205]
**days_to_freileg**	ARR	15	364 [280, 458]
**days_to_freileg**	ARR+II	72	162 [119, 212]
**days_to_freileg**	[Kruskal-Wallis]	149	KW *p* = 0.0000
**days_to_pros**	ARP	31	406 [326, 570]
**days_to_pros**	ARP+II	30	196 [136, 250]
**days_to_pros**	ARR	13	419 [356, 587]
**days_to_pros**	ARR+II	69	204 [151, 261]
**days_to_pros**	[Kruskal-Wallis]	143	KW *p* = 0.0000

**Table 5:** Treatment × operator (counts).

Treatment	Non-OMFS	OMFS/MKG
**ARP**	13	20
**ARP+II**	30	2
**ARR**	6	10
**ARR+II**	68	9

**Table 6:** Chi-square association and Cramér’s V.

Chi-Square	df	*p*	Cramer’s V
**46.363**	3	0.0	0.542

**Table 7:** Complication rate by operator (Wilson 95% CI).

Operator	N	Events	Rate	CI95_Lo	CI95_Hi
**Non-OMFS**	126	7	0.056	0.027	0.11
**OMFS/MKG**	41	0	0.0	0.0	0.086

**Table 8:** Pairwise Fisher tests (BH–FDR).

Pair	p_Raw	p_adj_BH
**Non-OMFS vs. OMFS/MKG**	0.196	0.196

**Table 9:** Ridge-penalized logistic regression (operator effects adjusted).

Term	Coefficient	OR	Note
**const**	−2.22	0.109	Ridge-penalized logit; CIs omitted due to quasi-separation.
**operator_OMFS/MKG**	−0.944	0.389	Ridge-penalized logit; CIs omitted due to quasi-separation.
**treatment_base_ARR**	0.0	1.0	Ridge-penalized logit; CIs omitted due to quasi-separation.
**immediate_ii**	0.0	1.0	Ridge-penalized logit; CIs omitted due to quasi-separation.
**age_at_op_years**	−0.009	0.991	Ridge-penalized logit; CIs omitted due to quasi-separation.

**Table 10:** Timelines by operator (median [IQR]; Kruskal–Wallis).

Metric	Operator	N	Median [IQR]
**days_to_freileg**	Non-OMFS	108	156 [118, 229]
**days_to_freileg**	OMFS/MKG	41	364 [236, 482]
**days_to_freileg**	[Kruskal-Wallis]	149	KW *p* = 0.0000
**days_to_pros**	Non-OMFS	107	210 [150, 290]
**days_to_pros**	OMFS/MKG	36	460 [337, 582]
**days_to_pros**	[Kruskal-Wallis]	143	KW *p* = 0.0000

**Table 11:** Pairwise Mann–Whitney (days to Freilegung, BH–FDR).

Metric	Pair	p_Raw	p_adj_BH
**days_to_freileg**	Non-OMFS vs. OMFS/MKG	0.0	0.0

**Table 12:** Pairwise Mann–Whitney (days to prosthesis, BH–FDR).

Metric	Pair	p_Raw	p_adj_BH
**days_to_pros**	Non-OMFS vs. OMFS/MKG	0.0	0.0

## Data Availability

The original contributions presented in this study are included in the article. Further inquiries can be directed to the corresponding author.
